# Resonant song recognition and the evolution of acoustic communication in crickets

**DOI:** 10.1016/j.isci.2024.111695

**Published:** 2024-12-26

**Authors:** Winston Mann, Bettina Erregger, Ralf Matthias Hennig, Jan Clemens

**Affiliations:** 1 ENI-G, a Joint Initiative of the University Medical Center Göttingen and the Max Planck Institute for Multidisciplinary Sciences, Göttingen, Germany; 2 University of Natural Resources and Life Science, Vienna, Austria; 3 Humboldt-Universität zu Berlin, Department of Biology, Berlin, Germany; 4 Department of Neuroscience, Faculty VI, University of Oldenburg, Oldenburg, Germany

**Keywords:** Bioacoustics, Entomology, Neuroscience

## Abstract

Cricket song recognition is thought to evolve through modifications of a shared neural network. However, the species *Anurogryllus muticus* has an unusual recognition pattern that challenges this view: females respond to both normal male song pulse periods and periods twice as long. Of the three minimal models tested, only a single-neuron model with an oscillating membrane could explain this unusual behavior. A minimal model of the cricket’s song network reproduced the behavior after adding a mechanism that, while present in the full network, is not crucial for song recognition in other species. This shows how a shared neural network can produce diverse behaviors and highlights how different computations contribute to evolution. Our results also demonstrate how nonlinear computations can lead to rapid behavioral changes during evolution because small changes in network parameters can lead to large changes in behavior.

## Highlights

• The cricket *Anurogryllus muticus* exhibits resonant song recognition behavior
• Minimal models cannot capture all aspects of the resonant behavior
• The song recognition network shared by all crickets reproduces the resonance
• Nonlinear neural computations support rapid behavioral evolution

## Graphical abstract

**Figure:**
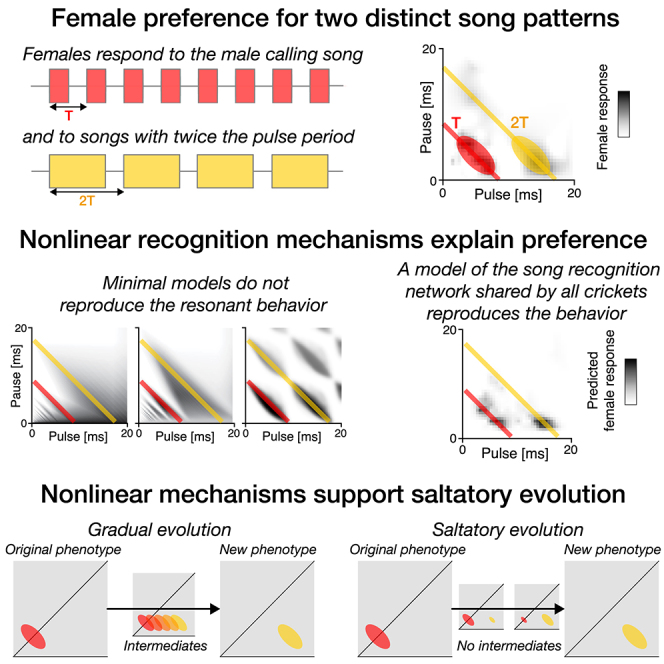

## Introduction

Evolution has given rise to diverse animal forms and behaviors. Much of this phenotypic diversity is shaped by the process of mate recognition and sexual selection, and various categories of phenotypic cues—visual, acoustic, chemical, and tactile—must be integrated for mate choice decisions to be made. For many species, acoustic signals—calling or courtship songs—are among the first features to be recognized and evaluated in mate choice decisions. The acoustic communication signals produced during courtship behaviors are therefore highly diverse and contribute to species recognition. However, how the neural networks that produce this behavioral diversity evolve is largely unknown. A common hypothesis is that novel behaviors arise from shared neural networks—mother networks—through small changes in connectivity and in cellular properties.^1^^,^^2^^,^^3^^,^^4^^,^^5^ At first sight, the idea of incremental changes in network parameters underlying behavioral evolution is at odds with the observation that behavior can change rapidly^6^^,^^7^^,^^8^^,^^9^ and outlier species—species with a highly unusual phenotype in a species group—challenge the mother network hypothesis. Evolutionary-developmental biology explains rapid morphological change—so-called "hopeful monsters"—through the re-use and modification of nonlinear gene-regulatory modules.^10^^,^^11^ Because of these nonlinearities, large morphological changes can then arise from a single mutation in a saltatory instead of a gradual manner. Similarly, behavioral innovations—"behavioral monsters"—could emerge from small changes in a neural network from the nonlinear mapping between the network’s parameters and the behavior.

Experimental tests of the mother network hypothesis are challenging because they involve characterizing and comparing the network properties across many species in a group and then causally linking the changes in network properties to changes in behavior. However, a precondition for the mother network hypothesis is that the shared network has the capacity to produce the diverse species-specific behaviors in a group. Computational modeling can help assess this capacity from behavioral data, by comparing the observed behavioral diversity with that produced by a computational model of the shared mother network. If the model of the proposed mother network fails to reproduce the behavior of a specific species then that species likely has undergone more drastic changes in its recognition mechanism inconsistent with the mother network hypothesis. Conversely, the hypothesis is supported if the network can reproduce all observed behaviors, including those of the "behavioral monsters"—species with unusual behavioral phenotypes.

**Figure 1:**
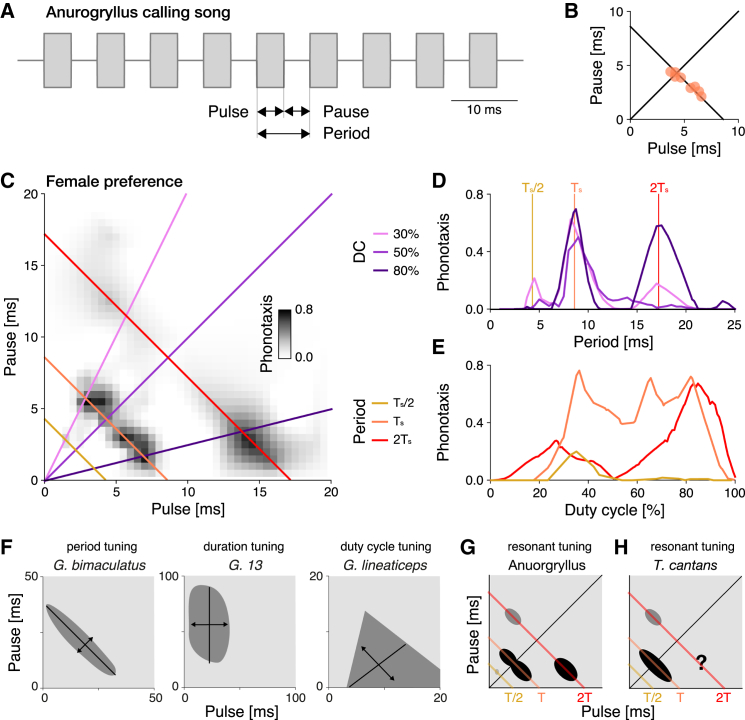
Anurogryllus is a cricket species with resonant song recognition (A) Schematic of the calling song of males from the cricket species *Anurogryllus muticus* (from now referred to as Anurogryllus). The song consists of a train of pulses with a specific pulse duration and pause. The period is the sum of pulse and pause and corresponds to the song’s rhythm. The duty cycle (DC) is the percentage of the period occupied by the pulse and corresponds to the song’s energy. (B) Pulse and pause parameters from eight Anurogryllus males. The diagonal line corresponds to a DC of 50%, the anti-diagonal to the average pulse period $T_{s}=8.6$ ms. See Table 1 for all song parameters. (C) Female phonotaxis for pulse trains with different duration and pause parameters visualized as a pulse-pause field (PPF). Phonotaxis is color coded with darker greys representing stronger phonotactic responses (see color bar). Diagonal lines indicate stimuli with DCs of 30, 50, and 80%, shown in D as the phonotaxis along these diagonals. The anti-diagonal lines show transects with constant period stimuli shown in E at the average pulse period of the male song $T_{s}$ (orange), at half ($T_{s}/2$, yellow), and twice ($2T_{s}$, red) the song period. Females respond strongly to pulse patterns with the period of the males’ song, but also at twice that period, indicating resonant song recognition. See Table S1 for the statistical significance of the individual peaks. The PPF was obtained by the interpolation of the average phonotaxis values measured for 75 artificial stimuli in 3–8 females (Fig. S1). (D) Period tuning as a function of DC given by three transects through the PPF in C (see legend in C). Vertical lines indicate $T_{s}/2$ (yellow), $T_{s}$ (orange), and $2T_{s}$ (red). (E) DC tuning as a function of song period, derived from transects through the PPF in C (see legend in C). (F) The three previously known female preference types for the pulse pattern of the male calling song in different cricket species: period (left), duration (middle), and DC (right). The solid black lines indicate the major or most tolerant axis that defines the tuning type, and the double sided arrows perpendicular to the major axis show the most sensitive feature axis. (G) Schematic of resonant recognition from Anurogryllus, simplified from C. (H) Resonant recognition from the katydid *Tettigonia cantans*.^12^ The question mark indicates the range of stimulus parameters not tested in the original study. Anti-diagonal lines in G and H indicate stimuli with $T_{s}/2$ (yellow), $T_{s}$ (orange), and $2T_{s}$ (red). See also Fig. S1 and Table S1.

We address the question of behavioral diversity and neuronal evolution in the context of acoustic communication in crickets. Males produce pulsed calling songs with species-specific pulse and pause durations ranging between 10 and 80 ms (Figures 1A and 1B).^13^ The songs are either produced in chirps consisting of a few pulses or continuously, in trills. Females evaluate the song on the timescale of pulse pause and duration, and of chirps/trills (Figure 1A).^14^ Attractive songs elicit positive phonotaxis in the female. The female tuning for the calling songs can be quantified by measuring the phonotactic behavior for artificial pulse patterns in a two-dimensional parameter space spanned by pulse and pause duration (Figure 1C). The strength of phonotactic orientation toward the acoustic stimulus then serves as a measure for the strength of recognition. So far, preference functions are known from 18 cricket species, and they all reveal unimodal preferences for a single continuous range of song features (^15^^,^^16^^,^^17^^,^^18^^,^^19^^,^^20^^,^^21^^,^^22^^,^^23^ and Ralf Matthias Hennig, unpublished data). The known preferences fall into three types, characterized by the females’ selectivity for specific features of the pulse song: Tuning for pulse duration, for period (pulse plus pause), and for duty cycle (duration divided by period, referred to from now on as "DC") (Figure 1F). Tuning for pause duration is a fourth possible phenotype, but this one has not yet been reported in crickets. Song recognition based on the duration or period of acoustic signals is not restricted to crickets but is found throughout the animal kingdom.^24^^,^^25^^,^^26^^,^^27^ Understanding the principles underlying the evolution of pulse song recognition in crickets can therefore inform similar studies in other species groups.

We have recently shown that the song recognition network described in the period-tuned *Gryllus bimaculatus* can produce the diversity of song recognition known in crickets. In *G. bimaculatus*, five neurons recognize the song in five steps^28^: 1) The ascending neuron 1 (AN1) pools and transmits to the brain information from auditory receptors in the prothorax and produces an intensity-invariant copy of the song pattern.^29^ 2) The local neuron 2 (LN2) receives input from AN1 and provides inhibition to LN5 and LN4. 3) The non-spiking LN5 produces a post-inhibitory rebound potential at the end of each song pulse. 4) LN3 fires only in response to coincident input from the rebound in LN5 and a delayed input from AN1. The input delay from AN1 is tuned such that coincidence only occurs for pulses with the species-specific period of 30 ms. 5) LN4 receives inhibition from LN2, which further sharpens the feature tuning. The tuning of LN4 for the pulse song matches that of the phonotaxis response. Similar principles of temporal pattern recognition with delay lines and post-inhibitory rebounds are known from many systems^30^^,^^31^^,^^32^ and understanding the capacity and constraints of this algorithm in crickets can therefore shed light on temporal pattern recognition across systems. A computational model reproduced the response dynamics of all neurons in this network as well as the behavioral output^33^ and revealed that the network from *G. bimaculatus* can produce the three preference types known in crickets—preference for period, pulse duration, and duty cycle—through changes in network parameters such as synaptic strengths or intrinsic neuronal properties. Thus, the *G. bimaculatus* network could be the mother network producing the diversity of song recognition in crickets.

We here describe the male song and female preference of the cricket species *Anurogryllus muticus* (from now on referred to as Anurogryllus). Anurogryllus females exhibit a multi-peaked recognition phenotype that is unreported in crickets and could challenge the hypothesis of a shared mother network: Females are attracted not only to the period of the male song but also to twice the period (Figures 1C–1E). Importantly, all other known cricket species have preference functions with a single peak (^15^^,^^16^^,^^17^^,^^18^^,^^19^^,^^20^^,^^21^^,^^22^^,^^23^ and Ralf Matthias Hennig, unpublished data) while Anurogryllus exhibits a multi-peaked preference function. All existing evidence, therefore, points toward Anurogryllus having a phenotype that is highly unusual and an outlier in the context of crickets, consistent with the concept of "behavioral monsters". Responses to multiples or fractions of a song’s period have only been shown in a single species of katydids, *Tettigonia cantans* (Figure 1H), and such responses are consistent with a resonant mechanism for song recognition.^12^ Computational modeling in katydids has suggested that delay-based mechanisms can not explain the resonant responses in the katydid and provided evidence for a nonlinear resonant-and-fire (R&F) mechanism of song recognition in katydids.^34^ Importantly, it is unclear whether the computational model of the song recognition network in crickets—which relies on a delay-based mechanism—can produce the resonant preference of Anurogryllus. Thus, Anurogryllus is a challenge to the mother network hypothesis and an opportunity to identify the computational principles that can give rise to resonant tuning.

Here, we provide further support for the mother network hypothesis, by demonstrating that it can produce the resonant recognition behavior of Anurogryllus. We first explore the tuning properties of minimal models of resonant behavior based on network and intracellular mechanisms and compare these results to those obtained from the full mother network model. Lastly, we explore the hypothesis that nonlinear computations, such as those that give rise to resonant recognition behaviors, could form the substrate for saltatory behavioral evolution.

## Results

### Anurogryllus exhibits an unusual resonant recognition phenotype

The calling song of Anurogryllus males consists of continuous trills with a pulse period $T_{s}$ of 8.5±0.3 ms, which corresponds to a pulse rate $f_{s}$ of 117.1±4.3  pulses per second (Figures 1A and 1B). This pulse rate is unusually high for cricket songs, which have pulse rates between 10 and 50 pulses per second.^35^ The song’s DC—given by the ratio of pulse duration and pulse period, and indicating how much of the song is filled by pulses—is 60 ± 10% (see Table 1 for a list of all song parameters). To quantify the preference of Anurogryllus females for the calling song we quantified the strength of the females’ phonotaxis response during the playback of 75 artificial pulse trains with different pulse and pause duration combinations (Figure 1C, S1). This confirms that females are attracted (perform positive phonotaxis) to the pulse trains produced by conspecific males: The two-dimensional preference function spanned by pulse duration and pause contains a broad peak covering periods of 8.5 ms and DCs of 33–80%, which overlaps with the distribution of male songs. This peak is partially split along the DC axis (Figure 1C).

**Table 1:** Parameters of the calling song of Anurogryllus males

Song parameter	Mean ± std	Range
Carrier frequency	7.0 ± 0.3 kHz	6.5–7.5 kHz
Pulse duration	5.1 ± 1.0 ms	3.7–6.6 ms
Pulse pause	3.4 ± 0.8 ms	2.1–4.4 ms
Pulse period	8.5 ± 0.3 ms	8.1–9.0 ms
Pulse rate	117 ± 4 pulses/s	111−124 pulses/s
Pulse DC	60 ± 10%	50−80%

Data from 8 males over 1 minute of song with at least 7500 pulses per male. Carrier frequencies from.^36^

However, the phonotaxis experiments also reveal that females are attracted to songs that differ substantially from the conspecific song and the tuning of these off-target responses implies a resonant recognition phenotype in Anurogryllus (Figures 1D and 1E; Table 1). These off-target responses appear at twice or half the song period: First, a song with twice the period of the male song (17 ms) with a high DC (90%) is almost as attractive as the conspecific song. Second, females are also weakly attracted to song with twice the conspecific period (17 ms) and lower DC (25%). Lastly, there is a weak and non-significant response peak at half the conspecific period (4.5 ms) and low DC (33%). The responses at integer fractions or multiples of the song’s fundamental rate indicate a resonant response mechanism. If we define $T_{s}=8.6$ ms as the period of the male song, and the fundamental rate $f_{s}=1/T_{s}=116$ pulses per second, then the weak peak at half the period, $T_{s}/2\approx 4.3$ ms, corresponds to the second harmonic, $2f_{s}$, while the peaks at twice the period, $2T_{s}\approx 17.2$ ms, corresponding to the second subharmonic, $f_{s}/2$.

This resonant song recognition behavior—with responses to three different types of pulse patterns—is unusual in a cricket (Figures 1F and 1G) but was previously shown in the katydid *Tettigonia cantans*^12^ (Figure 1H). The resonant phenotype in *T. cantans* is similar to that of Anurogryllus: *T. cantans* females are attracted to pulse trains with the period of the male song (period 40 ms, DC 50%), and to subharmonics of the male song—songs with twice the conspecific period (80 ms, DC 25%). *T. cantans* does not respond to harmonics (half the period, 20 ms) and it was not tested whether females are attracted to twice the period at higher DCs, the pattern that Anurogryllus is most responsive to apart from the conspecific song. A simple delay-line based mechanism in *T. cantans* was ruled out as a potential mechanism for resonance using experimental tests, but a resonate-and-fire neuron model with oscillatory membrane properties could reproduce the resonant song preference.^12^^,^^34^ Oscillatory neurons have therefore been proposed as a mechanism for song recognition in *T. cantans*. However, the rebound-based mechanism at the core of the song recognition network in crickets had not been considered, and it is unclear whether oscillatory neurons can reproduce the particular pattern of resonance observed in Anurogryllus.

### Simple models provide insight into the computational mechanisms of resonant tuning

The resonant phenotype in Anurogryllus challenges the mother network hypothesis, as the model of the song recognition network in crickets was only shown to produce all known single-peaked phenotypes, not the specific resonant phenotype of Anurogryllus^37^ (Figures 1F and 1G). We, therefore, tested whether this model network could also produce the resonant tuning of Anurogryllus. However, given that the computational model of the song recognition network in crickets is complex and has many parameters, we decided to first identify the computational principles and constraints that shape resonant tuning by investigating the ability of simple network and single-neuron models to qualitatively reproduce the resonant behavior of Anurogryllus. Simple models allow us to 1) isolate the minimal set of computations required for generating resonant behaviors, 2) facilitate the interpretation of the more complex network model, and 3) rule in or out alternative mechanisms not currently part of the mother network but that might be easily acquired during evolution. Given the simplicity of the models chosen, our goal was not a detailed reproduction of the Anurogryllus behavior (Figure 1C), but a reproduction of the most prominent properties of the period and DC tuning: namely the broad DC peak at the period of the male song, $T_{s}$, and the two response peaks at $2T_{s}$, with the dominant peak at high DC (Figure 1G).

We fitted three simple models to the behavioral data from Anurogryllus (Table 2): First, an autocorrelation model, which consists of a delay line and a coincidence detector.^12^ This is the simplest model that can produce resonances and shows that delays alone can produce resonant response peaks. Second, the rebound model, which is an extension of the autocorrelation model and captures the core computation of the mother network, in which the non-delayed input to the coincidence detector consists of offset responses from a post-inhibitory rebound.^28^^,^^37^ The rebound model will reveal whether the core computation of the mother network—a delay line, rebound, and coincidence detection—is sufficient to produce the resonant tuning of Anurogryllus. Lastly, we examined the resonate-and-fire (R&F) neuron, a single-neuron model with subthreshold membrane oscillations that reproduced the resonant behavior of *T. cantans*.^34^^,^^38^ This last model will allow us to examine how changes in intracellular properties, rather than network properties, can produce the resonant song recognition of Anurogryllus.

**Table 2:** Parameters of the simple models fitted to reproduce the Anurogryllus preference function

Model	Parameter name	Parameter value
Autocorrelation	delay $\Delta_{ac}$	17.0 ms
	output gain $g_{ac}$	0.21
Rebound	delay $\Delta_{rb}$	22.93 ms
	filter inhibitory gain $g_{i}$	0.045
	filter inhibitory duration $T_{i}$	5.06 ms
	filter excitatory gain $g_{e}$	0.1
	filter excitatory duration $T_{e}$	2.00 ms
Rebound with feedforward inhibition (remaining parameters were taken from the rebound model)	delay $\Delta_{ffi}$	7.29 ms
	filter inhibitory gain $g_{fi}$	1.01
	filter inhibitory duration $T_{fi}$	2.43 ms
	filter excitatory gain $g_{fe}$	0.63
	filter excitatory duration $T_{fe}$	2.45 ms
Resonate and fire	frequency $f_{rf}=\omega /2/\pi$	109.34 Hz
	damping b	−0.0005
	input gain $g_{s}$	0.027
	output gain $g_{rb}$	0.0025

#### Autocorrelation models produce resonant tuning but do not match the Anurogryllus behavior

**Figure 2:**
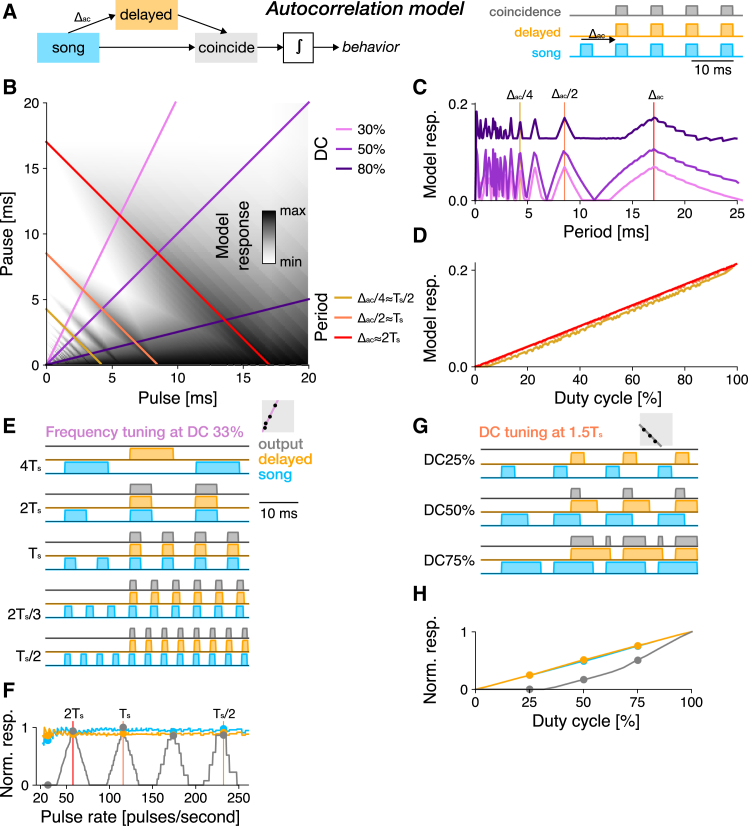
An autocorrelation model produces resonant tuning (A) In the autocorrelation model, a non-delayed (blue) and delayed (orange) copy of the stimulus are multiplied in a coincidence detector (gray). The output of the coincidence detector is integrated over the stimulus to predict the model response. The example traces show coincidence for a song with a pulse period that equals the delay $\Delta_{ac}$. (B) PPF for the autocorrelation model fitted to the preference data in 1C. Predicted response values are coded in greyscale (see color bar). Colored lines correspond to the DC and period transects shown in C and D (see legend). (C) Period tuning of the autocorrelation model for different DCs (see legend in B). Resonant peaks arise at even and odd fractions of the delay parameter $\Delta_{ac}\approx 2T_{s}$. Vertical lines indicate the pulse period transects shown in B. (D) DC tuning for three different pulse periods (see legend in B), corresponding to $T_{s}/2$, $T_{S}$, and 2T. DC tuning is high-pass for all periods. (E) Response traces from the autocorrelation model for songs with different periods (fractions and multiples of $T_{s}$) and a DC of 33%. Resonant peaks arise from coincidence at integer fractions (e.g., $1\Delta_{ac}/2=2T_{s}/3$) but not at multiples ($2\Delta_{ac}=4T_{s}$) of the delay parameter (stimulus–blue, delayed stimulus–orange, response–grey, see legend to the right). (F) Pulse rate tuning given by the integral of the stimulus (blue), the delayed stimulus (orange), and the response (gray) at 33% DC. Response peaks arise at integer multiples and fractions of $\Delta_{ac}$. Dots indicate pulse patterns shown in E. Vertical lines indicate the song periods shown in D. (G and H) Response traces for different DCs (25, 50, 75%) (G) and DC tuning (H) at a non-resonant pulse rate ($1.5T_{s}=12.9$ ms). Increasing the DC leads to coincidence even at this non-resonant pulse rate. Same color code as in E, F. Gray boxes in E and G illustrate the stimulus parameters for which traces are shown in the context of the PPF (compare B).

In an autocorrelation model, the song input is split into two pathways, one with a delay $\Delta_{ac}$, and one without a delay (Figure 2A). Responses from the delayed and non-delayed pathways are then multiplied in a coincidence detector that only responds when the delayed and the non-delayed inputs overlap in time. The model response is then taken as being proportional to the average output of the coincidence detector over the song.

The autocorrelation model fitted to the Anurogryllus data produces resonant response peaks for pulse rates at integer fractions, but not at multiples, of the delay $\Delta_{ac}$ (Figures 2B and 2C). The fitted value of $\Delta_{ac}=17$ ms corresponds to $2T_{s}$, the peak at twice the pulse period in the behavioral data (Figure 1E). Coincidence occurs if $nT=\Delta_{ac}$, leading to resonant peaks at periods that are fractions of the delay $T=\Delta_{ac}/n$ (or at pulse rates $f=n/\Delta_{ac}$) (Figures 2E and 2F). Thus, resonant peaks in the autocorrelation model arise at even and odd fractions of $\Delta_{ac}$ and coincide with $T_{s}$ and $2T_{s}$. However, the behavior only exhibits responses at even fractions of $\Delta_{ac}$. The lack of peaks at odd fractions of $\Delta_{ac}$ in Anurogryllus renders a pure autocorrelation-based mechanism for song recognition unlikely (Figure 1E).

Similar to the period tuning, the DC tuning of the fitted autocorrelation also does not match the behavioral data: The output of the autocorrelation model increases linearly with DC (Figure 2D), with maximal responses for constant tones without a pause (DC 100%). By contrast, Anurogryllus exhibits complex DC tuning with multiple peaks and, importantly, does not respond well to pulse trains with very high DCs (Figure 1E). The DC bias in the autocorrelation model arises because songs with longer pulses and shorter pauses are more likely to produce coincidence for any given delay (Figures 2F, 2G, 2H).

In sum, the autocorrelation model demonstrates that a delay is sufficient to produce resonance. However, autocorrelation alone is insufficient to qualitatively reproduce the pulse rate and DC tuning found in Anurogryllus.

#### A rebound mechanism suppresses responses to pulse trains with high duty cycles

**Figure 3:**
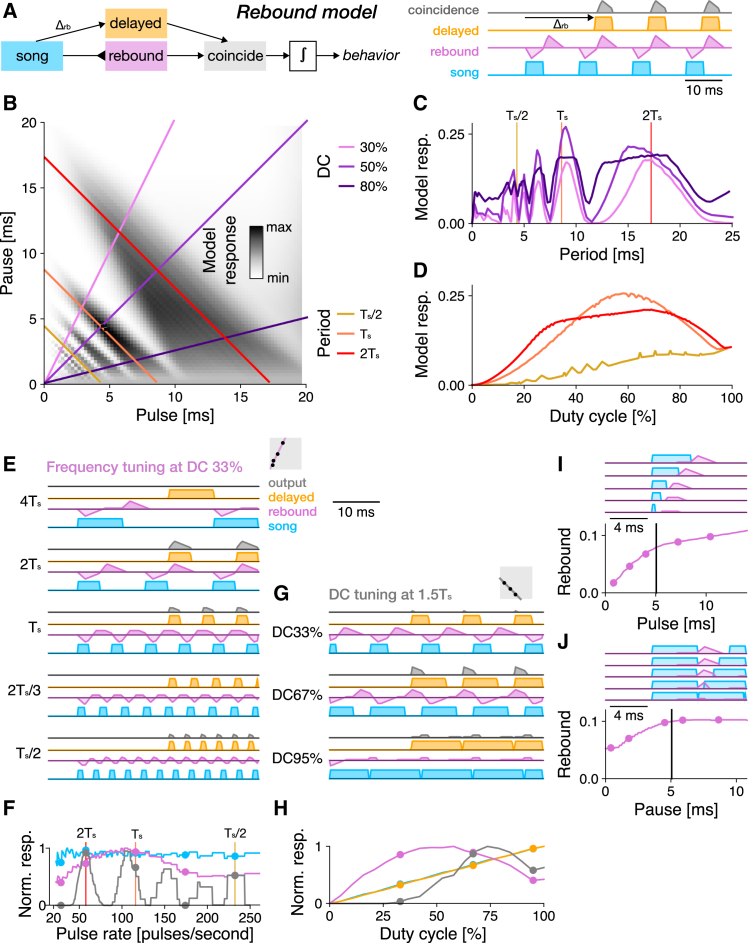
Tuning for pulse rate and duty cycle in the rebound model fitted to Anurorgryllus behavior (A) The rebound model is an extension of the autocorrelation model. The non-delayed branch (purple) is sign-inverted (blunt ended arrow indicates inhibition) and filtered by a bi-phasic filter to produce transient responses at pulse offsets that mimic a post-inhibitory rebound. The positive part of the rebound and the delayed stimulus are then combined through coincidence detection. (B) PPF for the rebound model fitted to the preference data in Figure 1C. Predicted response values are color coded (see color bar). Colored lines correspond to the DC and period transects shown in C and D (see legend). (C) Period tuning of the rebound model for different DCs (see legend in C). Vertical lines correspond to the pulse period transects shown in B. (D) DC tuning for three different pulse periods (see legend in C). DC tuning is high-pass for short periods ($T_{s}/2$, yellow) and band-pass for intermediate and long periods ($T_{s}$ (orange), $2T_{s}$ (red)). (E) Response traces of the rebound model for songs with different periods (fractions and multiples of $T_{s}$) and a DC of 33% (stimulus–blue, rebound response–pink, delayed stimulus–orange, response–grey, see legend to the right). (F) Pulse rate tuning given by the integral of the stimulus. Dots indicate periods shown in E. (G and H) Response traces for a DC sweep (33, 67, 95%) (G) and DC tuning (H) at a non-resonant period of $1.5T_{s}=12.9$ ms. Even at this non-resonant period, responses increase with DC, consistent with the broadening of the response peaks with DC in B and C. Responses decrease at very high DCs (short pauses), because the rebound is truncated by the next pulse (see J). Same color scheme as in E, F. (I and J) Integral of the rebound as a function of pulse duration (I) and pause (J). Dots in the curves (bottom) indicate example traces shown on top of each curve. A minimum pulse duration and pause duration (black lines) are required for the rebound to fully develop. At short pauses the rebound is interrupted by the following pulse (J). Gray boxes in E and G illustrate the stimulus parameters for which traces are shown in the context of the PPF (compare B).

The core computation for song recognition in the cricket *G. bimaculatus* is an extension of the autocorrelation model^28^^,^^37^ (Figure 3A): As in the autocorrelation model, the song is split into a delayed and a non-delayed path. The non-delayed path is then sign-inverted and filtered to produce transient responses at the end of each pulse, to mimic a post-inhibitory rebound. The rebound model produces outputs only when the delayed input coincides with the rebound.

The pulse rate tuning of the rebound model resembles that of the autocorrelation model, with resonant peaks arising close to even and odd fractions of the delay $\Delta_{rb}$ (Figures 3B and 3C, compare Figure 2C). However, the fitted value of $\Delta_{rb}=23ms$ matches neither multiples nor fractions of $T_{s}$. This is because the rebound is produced at the end of each pulse and coincidence therefore occurs if $n\cdot T+D=\Delta_{rb}$, where D is the pulse duration (Figures 3E and 3F). Resonant peaks occur at $T=\left(\Delta_{rb}-D\right)/n$ or $f=n/\left(\Delta_{rb}-D\right)$, close to even and odd fractions of $2T_{s}$ (Figures 3A, 3B, 3E, 3F). The responses to odd fractions of $T_{s}$ in the rebound model are not found in the behavioral data. Therefore, a pure rebound mechanism is unlikely to produce the Anurogryllus behavior.

The DC tuning of the rebound model is band-pass, with reduced but non-zero responses for continuous tones (high DC) (Figures 3D and 3G). This band-pass tuning arises from two opposing processes: On the one hand, responses increase with pulse duration up to a point set by the duration of the inhibitory filter lobe that produces the rebound. This is because the rebound is strongest if the pulse is long enough to saturate the rebound, which happens when it fully overlaps the inhibitory filter lobe (Figure 3I). However, a further increase in pulse duration at a fixed pulse period shortens the pauses and for short pauses, the rebound is interrupted by the next pulse (Figures 3G and 3J).

Overall, the rebound model fails to reproduce the qualitative features of the Anurogryllus responses. Period tuning exhibits excess peaks at odd fractions of the pulse rate as in the autocorrelation model. While the DC tuning is band-pass, as in Anurogryllus, responses to constant tones are still evident and the characteristic pattern with split peaks is missing. This failure to reproduce the Anurogryllus behavior is surprising given that the rebound constitutes the core mechanism of song recognition in crickets. However, we will show below that a rebound mechanism can produce the Anurogryllus behavior when combined with other computations found in the full network.

#### The resonate and fire model is a simple model that qualitatively matches the Anurogryllus behavior best

**Figure 4:**
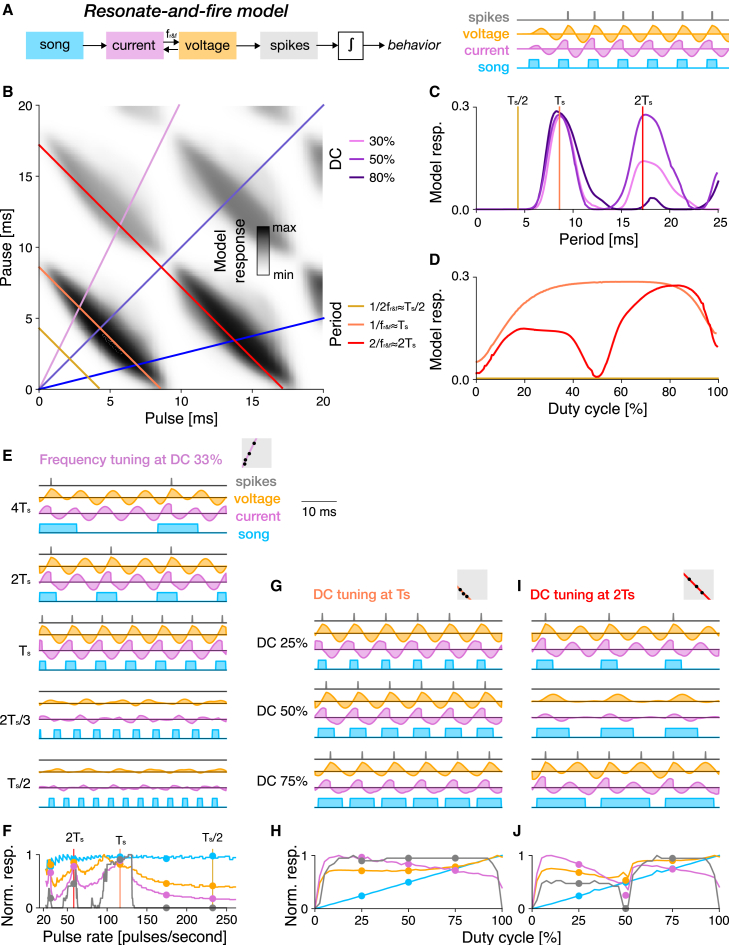
Tuning for pulse rate and duty cycle in the resonate and fire model fitted to Anurogryllus behavior (A) The resonate-and-fire (R&F) model is a spiking neuron model with bidirectionally coupled current (purple) and voltage-like (orange) variables. Inputs currents trigger oscillations with a frequency ω. Inputs are excitatory during positive phases and inhibitory during negative phases of the oscillations. If the voltage exceeds a threshold, a spike (gray) is elicited and the current and voltage are reset. (B) Pulse-pause field (PPF) for the R&F model fitted to Anurogryllus data. Colored lines correspond to the DC and period transects shown in C and D (see legend). (C) Period tuning of the R&F model for different DCs. Resonant peaks arise at integer multiples of $T_{s}$. The response at $2T_{s}$ is attenuated for lower DCs, as in the behavior. Vertical lines correspond to the periods shown in D. (D) DC tuning for three different pulse periods. There is no peak for $T_{s}/2$. At $T_{s}$, the DC tuning is band-pass. At $2T_{s}$, the DC tuning is bimodal, as in the data. (E) Response traces for the R&F model for songs with different periods (fractions and multiples of $T_{s}$) and a DC of 33% (stimulus–blue, current–pink, voltage–orange, spikes–grey, see legend). Membrane oscillations and responses are weak at fractions at $T_{s}$. Responses are strong at integer multiples of $T_{s}$. (F) Pulse rate tuning at DC 33%. Shown are the integrals of the stimulus (blue) and spiking response (gray). The current-like (pink) and voltage-like (orange) variables were rectified before integration. (G and I) Response traces for different DCs at $T_{s}$ (G) and $2T_{s}$ (I). (H and J) DC tuning at $T_{s}$ (H) and $2T_{s}$ (J). Dots mark the stimuli shown in G and I. DC tuning is unimodal at $T_{s}$ and bimodal at $2T_{s}$. Gray boxes in E, G, and I illustrate the stimulus parameters for which traces are shown in the context of the PPF (compare B).

As the last simple model, we fitted a resonate-and-fire (R&F) model to the Anurogryllus data. In contrast to the autocorrelation and rebound models, which are network models, the R&F model is a single neuron model that consists of coupled current and voltage-like variables (Figure 4A).^38^ This coupling leads to input-driven damped oscillations with a characteristic frequency $f_{rf}$. Inputs that arrive at positive/negative phases of the oscillation amplify/suppress this oscillation. If the voltage reaches a threshold, a spike is elicited and the current and voltage are reset. The R&F model can produce resonant behavior if the damping is weak and it was previously used to reproduce the resonant song recognition from *T. cantans*.^12^^,^^34^

The R&F model fitted to Anurogryllus data is weakly damped (less than 2% of the stimulus gain). It has a characteristic frequency $f_{rf}=109$  Hz, which translates to $T_{rf}=9.2$ ms—close to the pulse period of the Anurogryllus song. The R&F responds strongly if the incoming pulses hit the intrinsic oscillation during excitatory phases and resonant peaks therefore arise at multiples of the period $n\cdot T_{rf}=n/f_{rf}$. Thus, contrary to the autocorrelation and rebound models, the R&F model responds only to multiples (subharmonics), but not to fractions of $T_{rf}$ (harmonics) (Figures 4B and 4C). In the R&F, responses to fractions of $T_{rf}$ are suppressed, because inputs faster than $T_{rf}$ will arrive not only during the excitatory but also during the inhibitory phase of the intrinsic oscillation, reducing the net drive to the spike generator. By contrast, responses at multiples of $T_{s}$ exist because subsequent pulses will arrive during the excitatory phase of the membrane oscillation (Figures 4E and 4F). The R&F model reproduces the Anurogryllus responses at $T_{s}$ and $2T_{s}$ and, apart from excess responses at higher multiples of $T_{s}$, matches the behavior well.

The DC tuning of the R&F model is more complex than that of the autocorrelation and rebound models. At $T_{rf}$, the model responds with a single, broad peak to different DCs, whereas at $2T_{rf}$, two separate peaks—at high and low DCs—are visible (Figures 4B and 4D). The peak at the higher DC is greater than that at the lower DC, consistent with the Anurogryllus behavior. With this DC tuning, the R&F model qualitatively matches the Anurogryllus data best out of all models tested so far (Figure 1F). Note that the R&F also produces peaks at $3T_{rf}$, but stimuli covering these periods were not tested experimentally.

How does this complex DC tuning arise in the relatively simple R&F model? In the model, inputs during the excitatory phase of the membrane potential oscillation amplify the oscillation and therefore elicit spiking responses, while inputs during the negative phase suppress the spiking responses. Songs with a pulse period of $T_{s}$ match the period of the membrane oscillation and an input with a DC of 50% will produce the maximum output because it covers only the excitatory phase of the oscillations (Figures 1G and 1H). Shorter pulses (DC<50%) will produce weaker voltage responses because they engage the excitatory phase less, and longer pulses (DC>50%) will produce weaker voltage responses because they extend into the inhibitory phase. Pulse patterns with a pulse period of $2T_{s}$—twice the period of the oscillation—produce DC tuning with two broad peaks—around DC 25% and around DC 75%—and no responses at DC 50% (Figures 1I and 1J). The responses at DC 50% are suppressed because the pulse covers one full period of the oscillation, and therefore equally engages the excitatory and the inhibitory phases of the oscillation, resulting in weak spiking responses. Stimuli with smaller or larger DCs produce stronger responses because more of the excitatory phases of the oscillation are engaged. The peak at higher DCs is higher than that at lower DCs because the pulse hits the excitatory phase once per period for DCs below 50% and twice for DCs above 50%,^34^ as in (Figure 4I).

#### Simple network models, unlike the single neuron model, fail to reproduce the behavioral period and duty cycle characteristics

**Figure 5:**
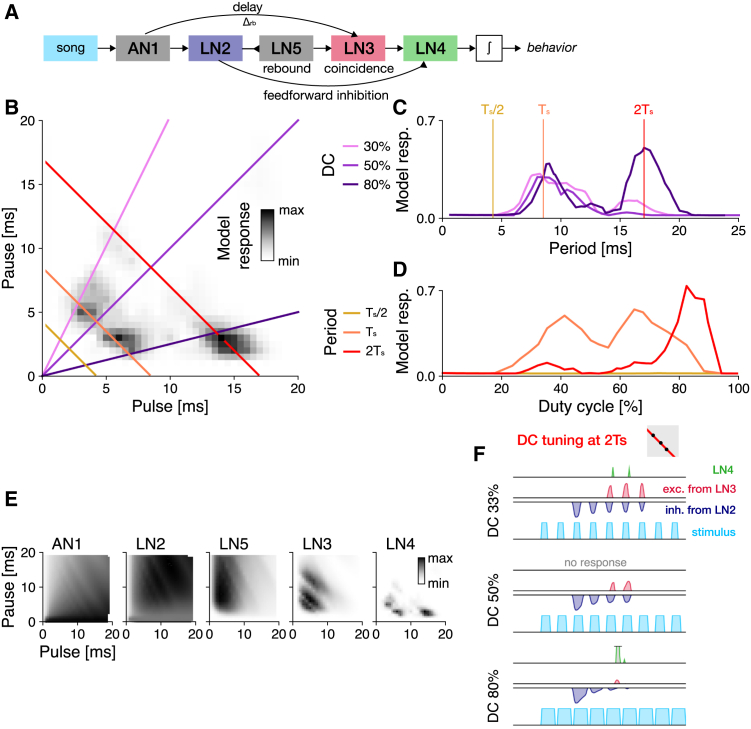
A model of the full song recognition network in crickets reproduces the resonant tuning of Anurogryllus (A) Schematic of the full 5-neuron network and internal connections. Pointy and blunt ended arrows indicate excitation and inhibition, respectively. Delay (AN1-LN3), rebound (LN5), and coincidence (LN3) are computations of the core rebound mechanism (Figure 3). Feedforward inhibition from LN2 to LN4 is crucial for reproducing DC tuning. (B) The resonant phenotype of Anurogryllus recovered with the five neuron model. Colored lines correspond to the period and DC transects in D and E. (C) Period tuning at 33%, 50%, and 80% DC, which each reveal the relative strength of the peaks at $T_{s}/2$, $T_{s}$, and $2T_{s}$. There is no response at the shortest period ($T_{s}/2$—yellow). At the period of male song ($T_{s}$—orange), DC tuning is band-pass. At the 17 ms period ($2T_{s}$—red), DC tuning is biphasic, as observed in the behavioral data. Vertical lines correspond to the DCs shown in C. (D) DC tuning for the different periods labeled in B, which shows that each peak has unique DC preferences. Compared with the behavioral data in Figure 1 which shows that Anurogryllus similarly demonstrates a bandpass preference around the male calling song $T_{s}$, and a preference for high DCs for the $2T_{s}$ peak. (E) Response profiles of the five neurons in the network. (F) Response traces for three songs (blue) along the $2T_{s}$ period transect at different DCs, showing the interaction of the excitatory coincidence detection output from LN3 (red) and the inhibition from LN2 (blue) to produce the output response in LN4 (green). The gray box in F illustrates the stimulus parameters for which traces are shown in the context of the PPF (compare B). See also Fig. S2.

Overall, none of the simple models were able to fully reproduce the Anurogryllus tuning. However, a single-neuron model—the R&F model—came closest, suggesting that changes in single neuron properties might underlie the emergence of resonant tuning in Anurogryllus (Figure 4). By contrast, simple delay-based models (autocorrelation and rebound) are insufficient to recover the Anurogryllus tuning (Figures 2 and 3): The delay-based models are resonant but they produce strong responses to very short periods (fractions of $T_{s}$) and are unable to replicate the DC tuning of Anurogryllus, in particular the double-peaked DC tuning at $2T_{s}$. Importantly, the failure of the rebound model, which replicates the hypothesized core mechanism of song recognition in crickets, challenges the mother network hypothesis^28^^,^^37^). However, the mother network, developed using electrophysiological data from *G. bimaculatus*, contains additional computations such as adaptation and feedforward inhibition. We therefore fitted a model of the full network, previously developed in^37^ (Figure 5A), to the behavioral data from Anurogryllus to test whether these additional computations can produce the behavior.

### The mother network can produce the resonant phenotype

A computational model of the song recognition network in crickets, that was originally constructed to reproduce electrophysiological data from *G. bimaculatus*,^37^ was fitted to the behavioral data from Anurogryllus females (Figure 1C, Table 3). This model reproduced the Anurogryllus behavior (5A): Resonant peaks at $T_{s}$ and $2T_{s}$, and DC tuning at $2T_{s}$ that is bimodal with a preference for higher DCs. This supports the mother network hypothesis—the network from *G. bimaculatus* can produce the preference profiles from all cricket species examined so far and could therefore constitute the shared network for song recognition in crickets. How does the characteristic period and DC tuning arise in the network? Above, we have shown that the rebound mechanism at the core of the network is sufficient to produce resonant responses but insufficient to produce the DC tuning at $2T_{s}$ (Figure 3D). We therefore investigated where in the network both response properties arise.

**Table 3:** Parameters of the 5 neuron "mother network" model fitted to reproduce the Anurogryllus preference function

Cell	Component	Parameters
AN1	Filter excitatory lobe	(Gaussian) width σ = 3.88, duration = 7.59 ms, input delay = 2.26 ms
	Filter inhibitory lobe	(Gaussian) width σ = 3.81, gain γ = 0.87, duration 293.04 ms
	Nonlinearity	(Sigmoidal) slope = 10.33, shift = 0.62, gain = 1.19, baseline = −0.29
	Adaptation	(Divisive normalization) timescale τ = 9999.93 ms, strength w = 85.75, offset x0 = 1
LN2	Input from AN1M	Delay = 7.59 ms, gain = 1.93
	Filter excitatory lobe	(Gaussian) width σ = 9.76, duration = 11.87 ms, gain = 0.59
	Filter inhibitory lobe	(Exponential) decay τ = 15.87 ms, duration N = 1000 ms
	Nonlinearity	(Rectifying) threshold = 0, gain = 4.22
LN5	Input from LN2	Delay = 13.13 ms, gain = 0.43
	Postsynaptic filter	(Differentiated Gaussian) width duration N = 8.94 ms, gain of the excitatory lobe = 0.41
	Postsynaptic nonlinearity	(Rectifying) threshold = 0, gain = 0.57
	Rebound filter excitatory lobe	(Gaussian) width τ = 0.02, duration = 5.18 ms, gain = −0.007
	Rebound filter inhibitory lobe	(Exponential) decay τ = 17.29 ms, gain = 6.5 duration N = 1000 ms
	Nonlinearity	(Rectifying) threshold = 0, gain = 0.006
LN3	Input from AN1	Delay = 16.59 ms, gain = 0.65
	Input from LN5	Delay = 9.67 ms, gain = 43.73
	Postsynaptic nonlinearity	(Rectifying) threshold = 0.24, gain = 6.82
	Adaptation	(Divisive normalization) timescale τ = 1463.98 ms, strength w = 0.16
	Nonlinearity	(Rectifying) threshold = 5.1, gain = 3.51
LN4	Input from LN2	Delay = 11.44 ms, gain = −58.26
	Input from LN3	Delay = 7.15 ms, gain = 3.75
	Nonlinearity	(Rectifying) threshold = −0.003, gain = 6.82

In the full network, LN3 is equivalent to the output of the simple rebound model as it is the coincidence detector that receives input from the rebound neuron LN5 and a delayed input from AN1. Accordingly, resonant tuning with responses at multiple periods in the network arises in LN3 (Figure 5E). Indeed, the effective delay between the two inputs to LN3 is 25.3 ms, similar to the delay $\Delta_{rb}=23$ ms found for the simple rebound model.

The DC tuning of Anurogryllus arises in the last neuron of the full network, LN4 (Figure 5E). LN4 receives excitatory input from the coincidence detector LN3 and feedforward inhibition from LN2. The inhibition from LN2 shapes the DC tuning by suppressing responses to song with a DC of 50% at $2T_{s}$ (Figure 5F): At DCs around 50%, the excitatory input from LN3 is ineffectual because it overlaps with the strong inhibition from LN2. For higher and lower DCs, inhibition is less potent and hence the output from coincidence detection prevails and LN4 responds. At lower DCs, inhibition is weak and offset in time from the excitatory input from LN3. At higher DCs, the excitation from LN3 is stronger and arrives slightly later than the inhibition. In summary, the Anurogryllus tuning arises serially, through two computations in the network model: Rebound and coincidence detection in LN3 shape the period tuning and feedforward inhibition from LN2 suppresses responses at wrong periods and shapes the DC tuning.

To confirm that a mechanism comprised of rebounds and feedforward inhibition is sufficient to reproduce the Anurogryllus behavior, we extended the simple rebound model (Figure 3) with delayed feedforward inhibition (Figure S2A). We used the parameters of the simple rebound model (Figure 3) and only fitted the delay and filter properties of the LN2-like input to LN4 (see Methods). This model is sufficient to reproduce the resonant period tuning (Fig. S2C) and the bimodal DC tuning of Anurogryllus (Fig. S2B–D). The DC tuning arises from the timing of excitatory and inhibitory inputs to LN4 (Fig. S2E), not from their strengths (Fig. S2F). Responses to a DC of 50% are suppressed in LN4 because excitation and inhibition arrive at the same time (Fig. S2E). For shorter/longer DCs, inhibition arrives too early/late to cancel the excitation.

### Nonlinear computations can accelerate the divergence of song preferences through saltatory evolution

Resonant, multi-peaked preference functions as found in Anurogryllus (Figures 1C–1E) may impair species discrimination, because they produce responses not only to the period of the conspecific song but also to its multiples or fractions. However, resonant recognition mechanisms could drive the fast co-divergence of song structure and song preference between sister species: According to the standard model of evolution, novel phenotypes evolve through an accumulation of small genetic changes that induce small phenotypic changes. However, an alternative, saltatory, model poses that nonlinearities in the mapping from genotype to phenotype can drive sudden large phenotypic changes.^39^ Evolutionary developmental biology has shown that strongly nonlinear developmental programs can give rise to morphological innovations from small genetic changes^11^—so-called morphological monsters. Resonant song recognition with responses to disjoint sets of songs is also the result of a highly nonlinear mapping from network parameters to behavior. If simple mechanisms existed to isolate individual resonant peaks, then behavioral preferences could jump between these peaks, resulting in sudden large changes in the female preference that will drive large changes in male song and a rapid isolation between sister species.

**Figure 6:**
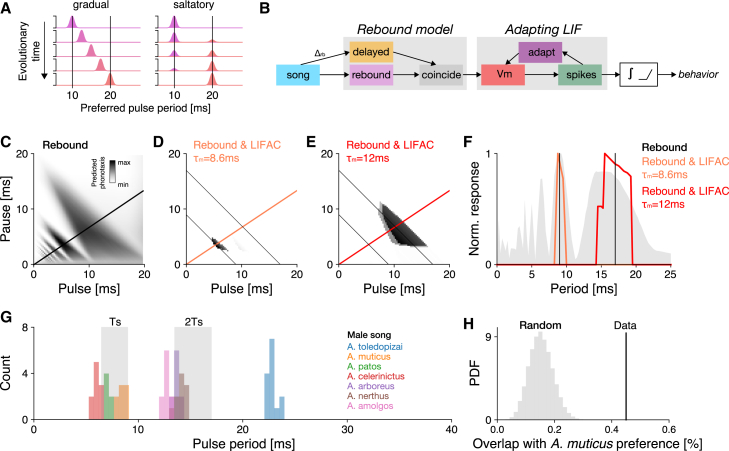
Resonances enable the saltatory evolution of song preferences (A) Evolution of the period preference (top to bottom) in a population under a gradual (left) and saltatory (right) mode. Under a gradual mode, small changes in the preference lead to a shift of the preference over time. Under a saltatory mode, the preference function of individuals jumps to a new peak and that new peak gets fixated without intermediates. (B) Structure of the rebound model with adaptation. The non-integrated output of the rebound model from Figure 3 was used to drive a leaky integrate and fire neuron with an adaptation current (LIFAC). The spike output of the LIFAC is then integrated to yield a value proportional to the phonotaxis. A rectifying nonlinearity (relu) is then used to further sharpen the tuning for the song. (C) PPF of the rebound model with resonant peaks used as the input to the LIFAC (same as Figure 3C). The two resonant peaks at ≈9 ms and ≈17 ms are shown as thin black anti-diagonal lines. The thicker black diagonal line shows the transect at a DC of 66% shown in F. (D and E) PPFs of the rebound-and-LIFAC model. The resonant peaks at ≈9 ms and ≈17 ms (thin black lines) were isolated by setting membrane time constants $\tau_{m}$ to 8.6 (D) and 12.2 m (E), respectively. The orange and red diagonal lines correspond to the transects at a DC of 66% shown in F. (F) Period tuning of the models in B–D for a transect through the PPF at a DC of 66%. (G) Distribution of song periods for seven Anurogryllus species. The gray shaded regions depict the responses of *A. muticus* females to the period of the male song ($T_{s}$) and to twice that period ($2T_{s}$) (cf. Figures 1C and 1D). (H) Overlap between songs from the 7 species and the resonant bands in the preference function of *A. muticus* females (gray bands in G) in the data (overlap 45%, black line) and under a random uniform model (gray histogram, 100,000 random samples, average overlap 15%). The observed overlap is unlikely to have arisen from that model ($p<10^{-12}$).

Spike-frequency adaptation (SFA) is one mechanism that can isolate individual peaks from a resonant preference function: SFA is ubiquitous in the nervous system and is also found in the song recognition network of *G. bimaculatus*.^28^^,^^29^^,^^37^ SFA in combination with the low-pass properties of the neuronal cell membrane results in a band-pass filter that can be tuned by changing the time constants of the membrane or of the adaptation current.^40^^,^^41^ We have implemented a simple proof-of-principle model to illustrate that SFA can isolate individual peaks from a resonant response field (Figures 6B–6F, Table 4). In the example, changes in the membrane time constant of an adapting neuron can change the relative amplitudes of the individual peaks and thus hide or reveal individual peaks without creating intermediate ones. This will exert selection pressure on the male song to jump to the new larger peak of the female preference function. SFA could thus be a mechanism through which acoustic communication evolves in a saltatory manner: Not by the gradual shifting of female preference and male songs but by jumping of the preferences and songs between relatively fixed resonant peaks (Figure 6A).

**Table 4:** Parameters of the model with spike-frequency adaptation

Period	Parameter name	Parameter value
Shared	Spike Refractory Period $\tau_{ref}$	1 m
	Adaptation Time Constant $\tau_{ada}$	5 m
	Adaptation Strength α	10 mV
	Spike Threshold $V_{thres}$	0.5 mV
4 m	Membrane Time Constant $\tau_{m}$	4 m
	Threshold $\theta_{relu}$	125 spikes
8 m	Membrane Time Constant $\tau_{m}$	8.8 m
	Threshold $\theta_{relu}$	72 spikes
16 m	Membrane Time Constant $\tau_{m}$	12 m
	Threshold $\theta_{relu}$	0 spikes

Resonant peaks at 4, 8, and 16 ms are isolated by adjusting the membrane time constant, $\tau_{m}$, and the threshold of the rectifying linear function, $\theta_{relu}$.

Direct evidence for this hypothesis is difficult to obtain as data on female song preference from other *Anurogryllus* species does not exist. However, given that female preference and male song are hypothesized to co-evolve and typically do so in crickets,^42^^,^^43^^,^^44^^,^^45^^,^^46^^,^^47^ song data could be used as indirect support of our hypothesis. Under the hypothesis, the pulse periods of males from different *Anurogryllus* species should be close to multiples or fractions of each other. While song data from the genus *Anurogryllus* is scarce we did find information on the songs of six other *Anurogryllus* species (Table 5), and that data is consistent with our hypothesis (Figure 6G). The distribution of song periods of all seven species is trimodal with the modes at periods of 7.2, 13.4, and 22.8 ms, close to integer multiples of each other. This is consistent with songs changing in integer steps along resonant peaks. In addition, the first two modes at 7.2 and 13.4 ms are close to the resonant bands of the *A. muticus* preference function (compare Figure 1D), with 45% of the song data overlapping with the resonant bands from *A. muticus* females. Under a uniform random model, the expected overlap is 15±4% and the observed distribution of songs is thus unlikely to have arisen by chance ($p<10^{-12}$, Figure 6H). While this preliminary analysis does not provide proof, the results are consistent with the hypothesis that song preference and song structure may develop in a saltatory manner by jumping between more or less fixed resonant peaks in *Anurogryllus*. In the future, a more comprehensive survey of male song and female preference in different *Anurogryllus* species is required to further test the hypothesis.

**Table 5:** Song periods and their distribution from seven Anurogryllus species

Species	Pulse period (mean ± std)	Sample Size (N)	Source
*A. muticus*	8.5 ± 0.3 ms	8	Erregger et al.^36^
*A. toledopizai*	22.8 ± 0.47 ms	14	Redü & Zefa^48^
*A. patos*	7.2 ± 0.22 ms	6	Redü & Zefa^48^
*A. celerinictus*	5.8 ±^a^ ms	23	Walker^49^
*A. arboreus*	13.5 ±^a^ ms	19	Walker^49^
*A. nerthus*	14.08 ±^a^ ms	unknown	Walker^50^
*A. amolgos*	12.65 ±^a^ ms	unknown	Walker^50^

a indicates that this statistic was not reported in the source literature.

## Discussion

In this article, we investigated the consequences of the multipeaked song recognition phenotype of Anurogryllus for the evolution of acoustic communication among crickets. Anurogryllus females respond to three different pulse patterns: pulse patterns matching the period of the male song but also to patterns with twice the period and low or high duty cycle (Figure 1). Using computational modeling, we tested whether this unusual recognition phenotype necessitates a novel recognition mechanism or if the hypothesized shared mechanism observed in other cricket species suffices.

First, to identify elemental computations required for resonant song recognition, we tested simple delay and filter-based network models, alongside a single-neuron model with resonant membrane properties (Figures 2, 3, and 4). While each model could resonate (produce responses with multiple peaks), it was the resonate-and-fire single-neuron model that best matched the tuning of Anurogryllus for both period and DC. That a single-neuron model qualitatively matches the behavior suggests that changes in intracellular properties capable of inducing oscillations of the membrane potential could underlie the resonant song recognition in Anurogryllus.

Critically, we found that a pure rebound mechanism, the core computation of the hypothesized shared mechanism for song recognition in crickets, is insufficient to reproduce the tuning of Anurogryllus and that additional computations present in the shared network are necessary (Figure 5): The core rebound mechanism gives rise only to the resonant period tuning but not the DC tuning, casting doubt on the mother network hypothesis. However, the addition of feedforward inhibition, present in the full model, recovered the DC tuning profile (Fig. S2). In *G. bimaculatus*, the cricket species in which the network was described, feedforward inhibition primarily served to refine period tuning,^28^ as no resonances appear at the coincidence detection stage of the network in this case. In Anurogryllus, it appears to have been coopted to modulate DC tuning by attenuating responses to intermediate DCs. This is in agreement with the fitted mother network model, in which the resonant response arises in two steps: The rebound-based mechanism at the core of the network shapes the period tuning, while feedforward inhibition shapes the DC tuning. Importantly, in the original network, the feedforward inhibition only sharpens the period tuning, suggesting that this computation can be re-used in Anurogryllus for another function. Overall, our study shows how novel behaviors can arise from the modification of existing intracellular and network computations.

### Mechanisms of resonant song recognition in Anurogryllus

In the absence of physiological recordings, computational modeling can be used to constrain hypotheses about the recognition mechanism of Anurogryllus. Here, we used two approaches: 1) Minimal models of networks and single-neurons, to identify the computations required to produce the Anurogryllus tuning, and 2) a complex network model based on the song recognition network from another species, *G. bimaculatus*, to test the potential of that network to produce resonant behavior. We identified two mechanisms that can give rise to the resonant song recognition in Anurogryllus: A cell-intrinsic mechanism based on oscillatory membrane properties (Figure 4). And a combination of two network mechanisms: rebound and feedforward inhibition (Figure 5 and S2).

The resonate and fire neuron, a single-neuron model that was previously used to reproduce resonant song recognition in a katydid^34^ qualitatively reproduced the pulse rate and DC tuning of Anurogryllus. It produced responses to $T_{s}$ and $2T_{s}$ and exhibited bi-modal DC tuning at $2T_{s}$ (Figure 4). Off-target responses in the model for longer periods could be suppressed by additional computations such as a high-pass filter, for instance via adaptation, a computation that is ubiquitous in the mother network.^33^^,^^41^ The resonant membrane properties could arise by changing the expression levels of specific ion channels in any of the neurons of the mother network, for instance, voltage-gated calcium (Ca_V_) or potassium (KCNQ, HCN) channels.^51^

We also found that the rebound mechanism in the mother network alone was not sufficient to produce the tuning of Anurogryllus (Figure 3). However, combining the rebound with feedforward inhibition recreates the period and DC tuning (Fig. S2). In the full network model (Figures 5A and 5B), these computations arise in different neurons of the network: First, the rebound produced by LN5 is combined with delayed excitation from AN1 in the coincidence detector LN3 to produce the resonant period tuning. Then, feedforward inhibition from LN2 shapes the DC tuning in LN4. Crucial for tuning the network are the response delays: from AN1 onto LN3 to tune the preferred periods^37^ and from LN2 onto LN4 to tune the DC responses (Fig. S2E, F). Ultimately, determining which of the two proposed mechanisms—single cell or network—generates the resonant behavior of Anurogryllus will require intracellular recordings that detect membrane oscillations and assess response delays.

### Resonances are rare because they are selected against, or because they have been missed in experiments

Our analysis of the simple models revealed that resonances can arise easily from common mechanisms such as delays or membrane oscillations (Figures 2, 3, and 4). However, multi-peaked response profiles appear to be rare.^12^ This may reflect selection against resonant tuning, because resonances broaden the female tuning to regions that fall outside of the male calling song, leading to the potential misidentification of mating partners. While multi-peaked tuning can still enable mate recognition if other signalers do not sing at the resonant off-target peaks,^52^ it is likely that these resonances are suppressed in many pattern recognition networks, for instance, through spike-frequency adaptation (Figures 6B–6F).

However, multi-peaked responses might also be underreported, since their detection requires a comprehensive and systematic sampling of the stimulus space when quantifying female preferences. Future playback experiments in crickets should therefore be designed to ensure the detection of resonances: Stimuli should not only densely sample different periods but should also do so at multiple DCs. For instance, a stimulus set that densely samples pulse periods at a DC of 50% would have missed the resonant peaks at twice the song period in Anurogryllus and *T. cantans* (Figures 1G and 1H). A characterization of the DC tuning also helps to differentiate between resonant mechanisms (Figures 2, 3, and 4): Only the R&F model produces bimodal DC tuning at $2T_{s}$, while autocorrelation and rebound mechanisms produce unimodal DC tuning. Sweep or chirp stimuli commonly used in electrophysiology have a changing pulse rate or period but are not sufficient for discriminating models since these stimuli have a constant DC.^53^ Similarly, the presence or absence of responses to odd and even multiples or fractions of the song period can disambiguate between different mechanisms (Figures 2, 3, and 4): The R&F model responds only to even multiples of the model’s characteristic period, while the simple delay-based models respond to both even and odd fractions. However, an interpretation of such experiments is complicated by the fact that the behavioral preference is the outcome of multiple computations, in the case of Anurogryllus possibly of a rebound mechanism combined with feedforward inhibition (Fig. S2).

### Resonant song recognition and the evolution of acoustic communication in crickets

Overall, our computational approach revealed the capacity of neural networks for change: The song recognition network described in *G. bimaculatus* consists of a set of elementary computations—rebounds, coincidence detection, adaptation, feedforward inhibition—that can give rise to a rich set of recognition behaviors. This network has the capacity to produce all recognition types known in crickets: For pulse pause, pulse period, DC, and even multi-peaked resonant tuning of Anurogryllus. This network could therefore serve as a mother network, that gives rise to the full diversity of song recognition in crickets. That even a small network, consisting of only 5 neurons can produce diverse behaviors highlights the enormous potential of neural networks to produce evolutionary novel phenotypes.

While simple models of the core rebound, delay, and coincidence detection mechanism only partially recovered the characteristics of the resonant Anurogryllus behavior, insights from the full model revealed that the function of the pulse recognition network in crickets might include additional selectivity for duty cycle via feedforward inhibition, the inclusion of which enabled the simple rebound model to replicate the resonant pattern. These results further suggest that the function of the pulse pattern recognition network in crickets cannot be conceptualized merely as a rate detector, but that it may additionally select for the duty cycle characteristics of incoming song, necessitating the inclusion of feedforward inhibition in even a minimal model for song feature recognition networks in crickets (Figure S2). More generally, the capacity of neuronal networks to drive evolutionary change stems in part from the multitude of nonlinear computations at the network and single-neuron level, which can be coopted to produce new behaviors.

### Nonlinear computations can drive saltatory behavioral evolution

The resonant mechanism for song recognition in crickets studied here is just one example of the many nonlinear computations inherent in the neuronal networks that drive behavior. However, they help illustrate a different view on the evolution of behavior: While the standard model of evolution, gradualism, assumes phenotypic change through the accumulation of small adaptive changes, an alternative view poses that large, saltatory change can drive rapid phenotypic change that is then fixed through selection.^10^^,^^39^ This saltatory model is supported by the existence of so-called "morphological monsters," such as flies with legs instead of antennae, which are a symptom of the highly nonlinear genetic networks and programs that drive morphological development.^11^ We propose that the highly nonlinear neuronal computations inherent in the brain can also drive saltatory behavioral evolution, and thus behavioral monsters: Animals with highly unusual behaviors. A saltatory mode of evolution may be most advantageous if rapid behavioral changes are adaptive, for instance in traits that support species evolution. The potentially multi-peaked recognition phenotypes driven by resonant mechanisms can be a trait that allows saltatory changes in song recognition. As one example, we have shown that spike-frequency adaptation after a resonant recognition network can support this scenario (Figures 6B–6F).^40^^,^^41^ Changes in adaptation parameters can suppress one resonant peak and amplify another, driving a switch to a novel preferred pulse period without intermediates (Figure 6A). Following the change in female preference will force male songs to change drastically as well. It is an open question what nonlinear mechanisms might support the rapid—and possibly saltatory—change of song pattern in the song pattern generators. However, the same mechanism at work in the female recognition network that gives rise to resonances—post-inhibitory rebounds, response delays, adaptation, inhibition—can also be found in the song pattern generators.^54^^,^^55^^,^^56^

While the idea of saltatory evolution is theoretically intriguing, it also generates testable hypotheses on the statistics of male song patterns and female song preference within a species group. Assuming that the male’s pulse pattern and the female preference co-evolve,^42^^,^^43^^,^^44^^,^^45^^,^^46^ saltatory evolution by jumping between fixed resonant peaks would lead to multimodal distributions of song parameters and song preferences in closely related species. While by no means ultimate since it is based on a small set of song data, our preliminary finding that the songs of seven species in the genus *Anurogryllus* are trimodally distributed is consistent with this prediction. To further test our hypothesis, songs from more species under the consideration of phylogenetic data are required. As is female preference data, which would show that females from different species also show either multi-peaked responses such as Anurogryllus (Figures 1C and 1D) or fall on a small number of peaks. In addition, if a species is "caught in the act" of transitioning from one peak to another, we would expect bimodal distributions of female preferences and/or male song parameters.

Given the existence of nonlinear computations in neural networks, the potential for saltatory behavioral evolution exists in every system and it might drive evolution whenever sudden phenotypic changes are adaptive.

### Limitations of the study

Our conclusions are based on computational modeling of behavioral data from a single species. Determining the song recognition mechanism in *A. muticus* will require intracellular recordings that detect membrane oscillations or assess response delays. In addition, a more comprehensive survey of male song and female preference in different *Anurogryllus* species is required to test whether resonant song recognition is common in the genus and to provide proof of saltatory evolution.

## Resource availability

### Lead contact

Further information and requests for resources should be directed to and will be fulfilled by the lead contact, Jan Clemens (jan.clemens@uol.de).

### Materials availability

This study did not generate new unique reagents.

### Data and code availability

• Data has been deposited at Github at https://github.com/janclemenslab/anurogryllus-resonance and are publicly available as of the date of publication at https://doi.org/10.5281/zenodo.14296669.
• Code has been deposited at Github at https://github.com/janclemenslab/anurogryllus-resonance and are publicly available as of the date of publication at https://doi.org/10.5281/zenodo.14296669.
• Any additional information required to reanalyze the data reported in this article is available from the lead contact upon request.

## Acknowledgments

We thank the following students who ran the behavioral experiments for establishing the Anurogryllus female response: Sofia Hayden, Daria Ivanova, Eileen Gabel, Kolja Haβ, Anne Görlitz. Funded by the 10.13039/501100001659Deutsche Forschungsgemeinschaft (DFG, German Research Foundation) as part of the SPP 2205, project number 430158535.

## Declaration of interests

The authors declare no competing interests.

## Author contributions

(1) Conceptualization - WM, MH, and JC.
(2) Animals and behavioral experiments - BE and MH.
(3) Modeling and analysis – WM and JC.
(4) First draft - WM and JC.
(5) Feedback on draft - BE and MH.

## STAR★Methods

### Key resources table

**Table:** 

REAGENT or RESOURCE	SOURCE	IDENTIFIER
**Deposited data**		

*A. muticus* song data	Erregger et al., 2017^36^	N.A.
*A. toledopezai* and *A. patos* song data	Redü et al., 2017^48^	N.A.
*A. celerinitctus* and *A. arboreus* song data	Walker, 1973^49^	https://orthsoc.org/sina/491a.htm,https://orthsoc.org/sina/492a.htm
*A. nerthus* and *A. amolgos* song data	Walker, 2015^50^	https://ufdc.ufl.edu/IR00007240/00002/downloads, https://ufdc.ufl.edu/IR00007240/00007/downloads

**Experimental models: Organisms/strains**		

*Anurogryllus muticus*	Erregger et al. 2018^57^	N.A.

**Software and algorithms**		

Code for running the computational models	https://doi.org/10.5281/zenodo.14296669	https://github.com/janclemenslab/anurogryllus-resonance
LabVIEW 7	National Instruments	https://www.ni.com/en.html
MetPy	Unidata	https://github.com/Unidata/MetPy

### Experimental model and study participant details

Behavioral experiments were performed with *Anurogryllus muticus* from the same colony as used in.^57^ The progeny of individuals caught on Barro Colorado Island in Panama were reared to adulthood at the Department of Zoology at the University of Graz in Austria and held at 25–28°C with *ad libitum* food and water. Starting with the second or third instar, individuals were separated from the colony and placed in individual plastic boxes.

### Method details

#### Male song recording and analysis

Individuals were placed in an array of separate boxes (mean temperature 24.9 ± 1.0°C SD) for a duration of 16–24 hours. Each box was equipped with a microphone and isolating foam to ensure acoustic isolation. Using customized software (LabVIEW 7, National Instruments, Austin, TX, USA), the microphone (TCM 141 Conrad; Conrad Electronic, Germany) in each box was scanned for 800 ms at a time with a sampling rate of 100 kHz and a male was recorded for 20 s if it produced sound during that 800-ms interval.^22^ The song carrier frequency was determined from the spectral peak of the raw waveform signal. For analysis of the temporal pattern, the normalized envelope of the song signal was computed after signal rectification by squaring and low-pass filtering at 200 Hz (equivalent to a temporal resolution of 2.5 ms). Temporal parameters such as pulse and pause duration were calculated when the envelope crossed or fell below a threshold value at 10–15% of the signal envelope.

As a preliminary test of our hypothesis that resonance might drive saltatory evolution, we examined the pulse periods of calling songs from seven more species of the genus *Anurogryllus* from the literature (Table 5). Aside from *A. muticus*, only *A. toledopizai* and *A. patos* included individual specimen measurements. For each of the species for which which only one pulse period was reported (*A. celerinictus, arboreus, nerthus, amolgus*), distributions of 10 songs were generated by sampling from a Gaussian with the species’ pulse period as the mean and the standard deviation from *A. muticus*.

Together with the species with individual specimen measurements, we find that roughly 45% of these 68 songs fall within either the $T_{s}$ or $2T_{s}$ period harmonics of *A. muticus* ($6.5\leq T_{s}\leq 9$ ms or $13.5\leq 2T_{s}\leq 17$ ms). To test whether this distribution of songs deviates from a random uniform distribution we simulated 100,000 trials in which 68 random songs drawn were from a uniform distribution between 0 and 40 ms and calculated for each of the 100,000 random distributions their overlap with the $T_{s}$ or $2T_{s}$ harmonic bands from *A. muticus*. The resulting distribution of 100,000 probabilities was then compared to the observed overlap of 45% and the probability of obtaining an overlap of 45% or greater was estimated by approximating the random distribution as a Gaussian to be $p<10^{-12}$.

#### Female preference functions

Female preference was tested using a trackball system as described in.^22^ Females, mounted to a metal rod, were placed on a hollow Styrofoam sphere (diameter: 100 mm, weight: 1.2–1.8 g) supported by an air stream between two perpendicularly placed loudspeakers (Piezo Horntweeter PH8; Conrad Electronic) in a wooden box with sound absorbing foam. Each loudspeaker was calibrated with a Bruel and Kjaer 2231 sound level meter and a half-inch condenser microphone (Bruel and Kjaer 4133 relative to 0.02 mPa, fast reading) at the top of the sphere where the female cricket was placed during experiments.

Digitally stored sound signals were transmitted from a hard disk by a D/A-board (update rate: 100 kHz, PCI 6221; National Instruments, Austin, TX, USA) to a digitally controlled attenuator (PA5; Tucker-Davis, Alachua, FL, USA), amplified (Raveland; Conrad Electronic) and broadcasted through the speakers. The longitudinal and lateral movements of the sphere were recorded by either a single optical sensor (Agilent ADNS-2051; Agilent Technologies, Santa Clara, CA, USA) at the bottom of the half-sphere or by two sensors (ADNS-5050; Avago Technologies, San Jose, CA, USA) with a focusing lens positioned laterally at an angle of 90.

A silent control was used to monitor baseline walking activity, and a continuous tone was used to control for motivation and selectivity of female responses. At the beginning and the end of each test session, a species-specific, attractive song signal was presented to control for possible changes in phonotactic motivation during a session. For each test signal, the lateral deviation of a female during signal presentation for each of the two speakers was averaged and normalized with respect to the attractive control signal. The resulting phonotactic scores were therefore typically between 0 (no orientation towards the sound signal) and 1 (strong orientation towards the signal), although negative scores (orientation away from the signal) and scores higher than 1 (orientation towards signal stronger compared to control stimulus) were possible. Test signals and controls were presented at 80 dB sound pressure level. All tests included the four control stimuli (silent, continuous tone, and an attractive stimulus at the beginning and end of a test) and eight test stimuli (total duration was 29 min per test), and were performed at 24°C.

Phonotaxis values were measured for 75 artificial pulse trains, split into 10 playlists. Each playlist was tested with 3-8 females and the phonotaxis values for each stimulus were averaged over the females (Fig. S1). All stimulus parameters, phonotaxis values, and number of animals are listed in a supplemental data file. From the 75 average phonotaxis values, we generated a two-dimensional preference function using natural neighbor interpolation implemented in metpy (URL: https://github.com/Unidata/MetPy). The preference function covered pulse and pause durations between 0 and 20 ms, with a resolution of 0.1 ms. Negative phonotaxis values in the interpolated preference functions were set to 0.

#### Modeling

##### Stimulus and response data

Song pulses were constructed as rectangular boxes with an amplitude of 1. While natural pulse trains in Anurogryllus last for many seconds, the models tested here have dynamics on the timescale of a few tens of milliseconds. To speed up simulations, we therefore used pulse trains with a duration of 400 ms and omitted onset and offset transients when translating the model output to predicted phonotaxis (see below). The stimulus set contained pulse trains with all combinations of pulse and pause durations between 0–20 ms sampled on a grid with an interval of 0.5 ms, totalling ${\left(20/0.5\right)}^{2}=1600$ stimuli. As the fitting target, we used the two-dimensional preference function from Anurogryllus females obtained by interpolating the experimental phonotaxis values as described above, but on a grid with a step size of 0.5 ms.

##### Predicting phonotaxis score from model responses

The predicted phonotaxis score, p, is given by the average model response r(t) over the stimulus duration $D_{s}$, excluding the first 25 ms and the last 10 ms to reduce the impact of response transients: $p=1/\left(D_{s}-35ms\right)\int_{25ms}^{D_{s}-10ms}r\left(t\right)dt$.

##### Model fitting

The models were fitted using the Nelder-Mead method implemented in scipy.optimize.minimize, by minimizing the mean-squared error between the interpolated phonotaxis values from the data and the model response. If not stated otherwise, initial values for all parameters were set using a vector of initial conditions chosen manually to speed up fitting. Fits were run multiple times from slightly different initial conditions to avoid getting stuck in local minima. The presented parameter values are from models with the lowest error. The model parameters for the simple models are listed in Table 2 and for the full network in Table 3. The code and parameters for running all models can be found at https://github.com/janclemenslab/anurogryllus-resonance.

##### Autocorrelation model

In the autocorrelation model (Fig. 2A), the stimulus s(t) is delayed by $\Delta_{ac}$, $s_{\Delta }\left(t\right)=s\left(t-\Delta_{ac}\right)$. A coincidence detector then multiplies s(t) and $s_{\Delta }\left(t\right)$ and scales the result with a gain factor $g_{ac}$: $r\left(t\right)=g_{ac}\cdot s\left(t\right)\cdot s_{\Delta }\left(t\right)$. We did not add a nonlinearity to the output r(t), like a sigmoidal, prior or after integration, since it did not produce quantitatively different predictions during fitting. The autocorrelation model was simulated with a resolution of 10 kHz.

##### Rebound model

The rebound model extends the autocorrelation model by inverting and filtering one of the two paths the stimulus takes before coincidence detection to produce offset responses at the end of each pulse: $s_{F}\left(t\right)=\int_{0}^{T}-s\left(t-\tau \right)\cdot h\left(\tau \right)d\tau$. The filter h(τ) consists of two lobes, defined as rectangular windows: An inhibitory lobe with negative gain $g_{i}$ and duration $T_{i}$, followed by an excitatory lobe with positive gain $g_{e}$ and duration $T_{e}$. The positive response components in $s_{F}\left(t\right)$ corresponding to the rebound are isolated using a rectifying linear function: $s_{R}\left(t\right)=f\left(s_{F}\left(t\right)\right)$, where f(x)=0 if x≤0, and f(x)=x if x>0. The coincidence detector then multiplies $s_{R}\left(t\right)$ and $s_{\Delta }\left(t\right)$: $r\left(t\right)=s_{R}\left(t\right)\cdot s_{\Delta }\left(t\right)$. The rebound model was simulated with a resolution of 4 kHz to accelerate the fitting process.

##### Rebound model with feed-forward inhibition

The rebound model with feed-forward inhibition extends the simple rebound model by including an additional inhibitory connection to the basic rebound model following coincidence detection (See Fig S2A). The added inhibitory path from stimulus to output (LN4) contains a bi-phasic filter with rectangular negative and positive lobes (similar to the filter in the rebound model) and a delay. The negative components of the output of the bi-phasic filter were then used as an inhibitory input to an LN4-like output neuron. The LN4-like neuron combines the inputs from the coincidence detector and the feed-forward inhibitory paths. To obtain the predicted phonotaxis value for a given stimulus, the output of the LN4-like neuron was passed through a rectifying linear function with threshold $\theta_{relu}=0$ and a linear gain $g_{relu}=1$ and then integrated. When fitting this model, the parameters of the simple rebound model fitted previously were kept fixed and only the additional parameters for the feed-forward inhibition branch (the delay time and the gain and duration of the inhibitory and excitatory lobe) were adjusted.

##### Resonate-and-fire neuron

The resonate-and-fire model was implemented following^38^:

$$\frac{dx}{dt}=b*x-\omega *y+g_{s}*s\left(t\right)$$

$$\frac{dy}{dt}=\omega *x+b*y$$

where x is a current-like variable, y is a voltage-like variable, b is the damping factor, ω is the intrinsic frequency, s(t) is the song input and $g_{s}$ is the gain of the song input. If y exceeds the threshold $y_{threshold}=1$, a spike with amplitude $g_{rb}$ is elicited and current and voltage are reset to $x_{reset}=0$ and $y_{reset}=1$:

$$\begin{array}{c} y=\left\{ \begin{array}{cc} x=x_{reset}\text{and}y=y_{reset}, & \text{if}y\geq y_{threshold}\\ y, & \text{otherwise} \end{array}\right. \end{array}$$

The differential equations were numerically integrated using the Euler method with a time step of 0.1 ms.

##### Full model of the song recognition network in *G. bimaculatus*

To test whether the song recognition network from *G. bimaculatus* described in^28^ can reproduce the resonant behavior of Anurogryllus, we used the model of the network from.^33^ This model was fitted to reproduce the response dynamics and the tuning of all neurons in the network using electrophysiological recordings from *G. bimaculatus* for a large set of pulse train stimuli.^28^^,^^42^ The forty-five parameters in the network model were fitted using the Nelder-Mead optimization algorithm, by minimizing the mean-square error between experimental and predicted phototaxis values (see Table 3 for the fitted parameters) using the parameter values found for *G. bimaculatus* as a start point. Several rounds of optimization were required to converge on the given parameter set, with Gaussian-distributed noise added to all parameters at the start of the initial optimization rounds to avoid undesirable local minima. Model fitting often yielded models that reproduced the tuning of Anurogryllus with only transient responses at the onset of the pulse train. Given that Anurogryllus song lasts multiple seconds and elicits phonotaxis throughout, we deemed these solutions physiologically unrealistic. We therefore added the constraint that responses of AN1 in the model should spike throughout the stimulus for pulse trains with conspecific parameters.

##### Modeling jumps between resonant peaks with spike-frequency adaptation

To demonstrate that individual resonant peaks can be isolated from a resonant response field, we added to the rebound model fitted to the Anurogryllus data (Figure 3; Table 4) a leaky integrate and fire neuron with an adaptation current (LIFAC) using the code published with.^41^ The LIFAC model is driven by the non-integrated output of the rebound model and acts as band-pass filter, because it combines the low-pass properties of a cell membrane and high-pass properties from adaptation.^40^ The total spike output from the LIFAC model for each stimulus is passed through a rectifying linear function with threshold $\theta_{relu}$ and a linear gain $g_{relu}=1$, to compute the predicted phonotaxis value.

The LIFAC neuron responds to a current input I by increasing the membrane potential V from which an adaptation current A is subtracted:

$$\begin{array}{cc} \tau_{m}\frac{dV}{dt} & =-V+I-A\\ \tau_{ada}\frac{dA}{dt} & =-A \end{array}$$

with time constants of the membrane and of adaptation, $\tau_{m}$ and $\tau_{ada}$, respectively. If the voltage V reaches the spiking threshold $V_{thres}$, a spike is elicited, and V is reset to $V_{reset}$ and the adaptation current strength A is incremented by α:

$$\begin{array}{c} V=\left\{ \begin{array}{cc} V_{reset}\;\text{and}\;A=A+\alpha , & \text{if }V\geq V_{thres}\\ V, & \text{otherwise} \end{array}\right. \end{array}$$

Each spike initiates a refractory period $\tau_{ref}$, during which both V and A are fixed to their reset values.

### Quantification and statistical analysis

Statistical analysis was performed in Python using the SciPy statistics package. Details on determining the statistical significance of the peaks in the behavioral preference profile can be found in the Supplemental information in S1. P-values for each peak were obtained from a paired one-sided t-test. Details on the statistical methods used in our investigation of the saltatory evolution hypothesis using other members of the *Anurogryllus* genus can be found in Figure 6.
