# Supplementary material for: Resonant song recognition and the evolution of acoustic communication in crickets
Source: iScience. 2024 Dec 26;28(2):111695. doi: 10.1016/j.isci.2024.111695 (PMC11773217; doi:10.1016/j.isci.2024.111695)
Supplement: Document S1. Figures S1 and S2, Table S1 [file mmc1.pdf]

iScience, Volume *28*

Supplemental information

Resonant song recognition and the evolution of acoustic communication in crickets

Winston Mann, Bettina Erregger, Ralf Matthias Hennig, and Jan Clemens

Supplemental Information

Peak	Females tested	p-value
$T_s/2$ (4.5 ms)	7	0.058
T_s (8.5 ms)	7	0.006
$2T_s$ (17 ms), high DC	4	0.002
$2T_s$ (17 ms), low DC	7	0.007

Table S 1: Statistical tests for each peak in the Anurogryllus phenotype (Related to Fig. 1).
P-values were obtained from a paired one-sided t-test testing the hypothesis that the responses of the individuals to songs at the peak are greater than a silent control. All peaks, except for the peak at $T_s/2$, are significant. The broad peak at $2T_s$ for low DC was evaluated using two points within this peak for which different sets of females were tested. The stimuli for these low DC points have either a pause of 12.5 ms and a duration of 4.5 ms, or a pause of 11.2 ms and a duration of 5.8 ms. The high DC condition for $2T_s$ was evaluated at a pause of 2.8 ms and a duration of 14.2 ms.

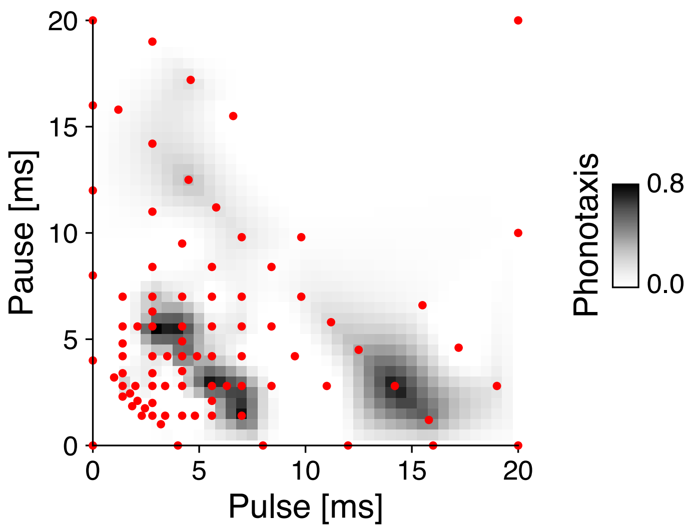

Figure S1: Pulse train stimuli used for estimating the pulse-pause field (PPF) (Related to Fig. 1).
Individual pulse trains for which phonotaxis values were measured are shown as red dots. The PPF (color coded, see color bar) was obtained by natural neighbor interpolation of the phonotaxis values on a dense 41x41 grid (all combinations of pulses and pauses between 0 and 20 ms with a step size of 0.1 ms). Phonotaxis values at the boundaries (pulse or pause 0 ms) were set to 0.

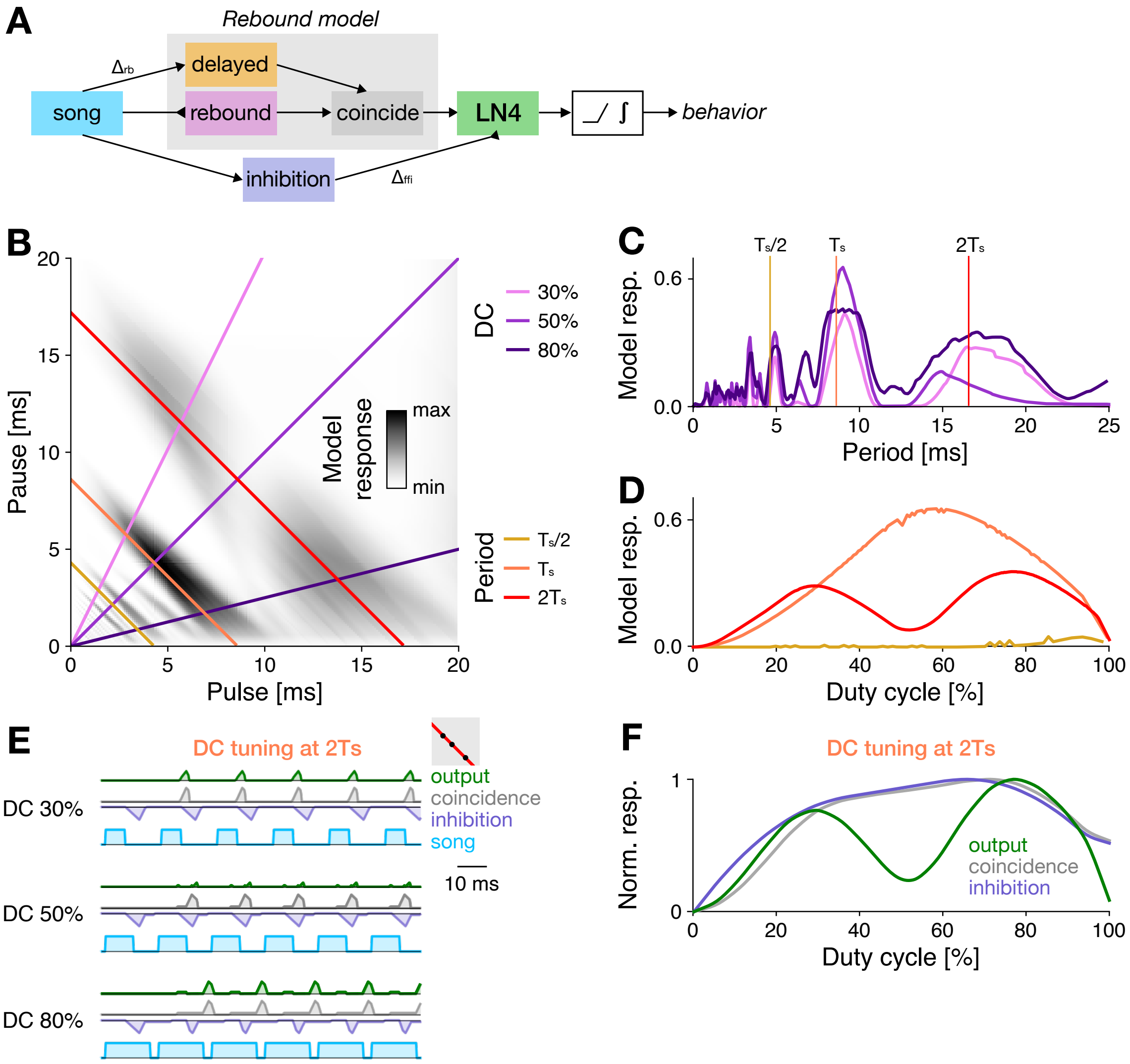

Figure S2: A combination of rebound and feed-forward inhibition are sufficient to create the tuning of Anurogryllus. (Related to Fig. 5).

A Schematic of the rebound model with delayed feed-forward inhibition. An LN4-like neuron receives input from the coincidence detector of a rebound model and from inhibition.

B PPF illustrating the responses produced by the modified rebound model fitted to behavioral data from Anurogryllus (Fig.1C, see color bar), demonstrating the restored bimodal shape of the 17 ms period transect. Colored lines correspond to the DC and period transects shown in C and D.

C Period tuning of the model for different DCs. Vertical lines correspond to the periods shown in D.

D DC tuning for three different pulse periods, corresponding to $T_s/2$, T_s, and $2T_s$. The curves indicate bandpass preference around the male calling song T_s, and bimodal DC tuning for the $2T_s$ peak.

E Example traces showing how the delay timing of inhibition (blue) interacts with the coincidence detection output (grey) to produce bimodal tuning along the $2T_s$ 17 ms period transect. Inhibition at 50% DC coincides with the timing of the coincidence detection output, fully suppressing responses.

F DC tuning of the rebound output (grey) vs the feed-forward inhibition (blue) for the 2T 17 ms transect, which produces the final bimodal tuning (green) as observed in the behavior data.
